# Feasibility of large-scale in silico transcatheter aortic valve implantation trials using a fast-to-evaluate model

**DOI:** 10.1007/s10237-026-02136-9

**Published:** 2026-09-25

**Authors:** Sabine Verstraeten, Marloes van Driel, Robin Willems, Sai Divi, Martijn Hoeijmakers, Frans van de Vosse, Clemens Verhoosel, Wouter Huberts

**Affiliations:** 1 Biomedical Engineering, Eindhoven University of Technology, Dominee Theodor Fliednerstraat 2, 5631 BN Eindhoven, The Netherlands; 2 Mechanical Engineering, Eindhoven University of Technology, Dominee Theodor Fliednerstraat 2, 5631 BN Eindhoven, The Netherlands; 3 Synopsys Inc, High Tech Campus 41, 5656 AE Eindhoven, The Netherlands; 4 Computational Science Lab, Faculty of Science, Informatics Institute, University of Amsterdam, Science Park 900, 1098 XH Amsterdam, The Netherlands

**Keywords:** In silico trials, Fast-to-evaluate models, Transcatheter aortic valve implantation, Paravalvular leakage

## Abstract

Although transcatheter aortic valve implantation (TAVI) has been demonstrated to be a successful treatment for aortic stenosis, it remains associated with complications, such as paravalvular leakage (PVL). To address these, TAVI devices continue to undergo iterative development. Integration of in silico trials into the regulatory validation pathway offers a promising approach to accelerate the development and clinical implementation of novel TAVI devices. This study addresses the feasibility of conducting large-scale in silico TAVI trials using a virtual cohort generator (VCG) combined with a fast-to-evaluate model. The objective is to investigate anatomical and procedural predictors of PVL in silico, as was done in an earlier clinical study. A virtual cohort of 500 synthetic aortic stenosis patients was generated, that matched anatomical and demographic characteristics of the clinical population. Using a novel fast-to-evaluate TAVI deployment model, nearly 29,000 simulations were performed across multiple model parameter combinations per patient. Shape and demographic distributions in the in silico trial, remained within the bounds of the clinical study. Among the investigated anatomical parameters, a higher angle between left ventricular outflow tract and ascending aorta was found in patients with significant PLV, in both clinical and virtual cohorts. Additionally, the relationship between PVL and implantation depth appeared highly patient-specific, which is in line with findings in clinical studies. The ability to systematically test multiple TAVI deployments scenarios per patient, which is unfeasible in clinical practice, provides valuable insights for procedure design and optimisation. Overall, the results support the feasibility of implementing large-scale in silico TAVI trials, using the VCG and a fast-to-evaluate model, into the regulatory validation chain.

## Introduction

**Fig. 1:**
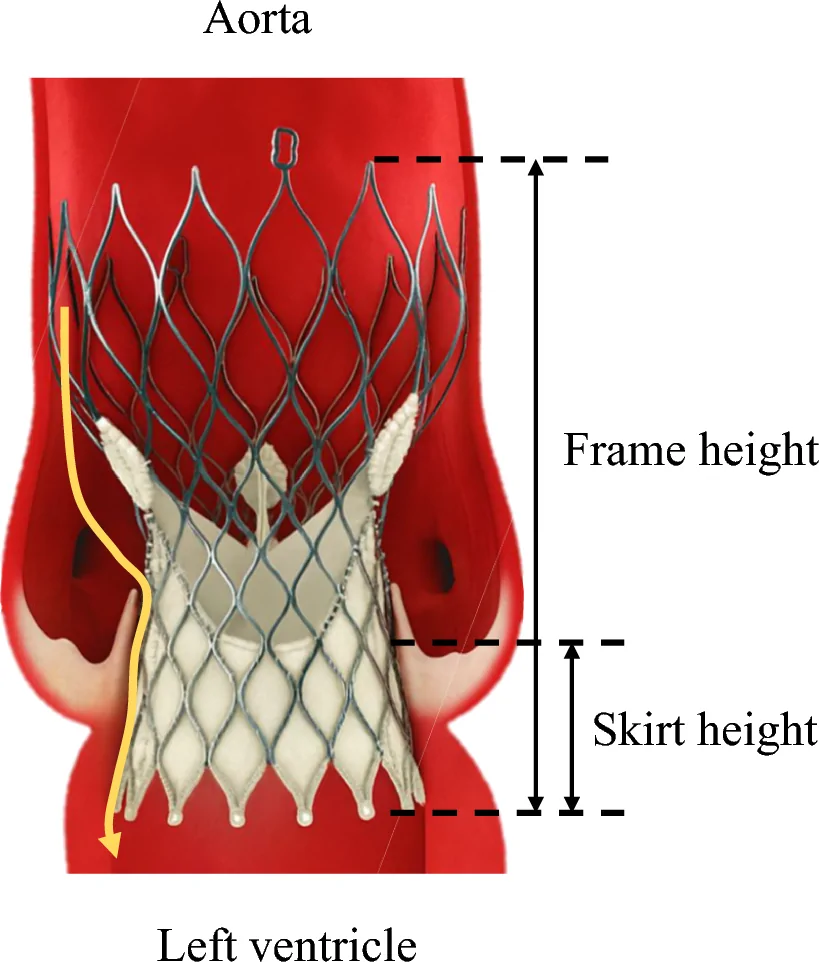
Schematic representation of a self-expandable TAVI valve, deployed in the native valve (adapted from Fornell (2014)). Frame and skirt heights are indicated. Paravalvular leakage is depicted with the yellow arrow

Transcatheter aortic valve implantation (TAVI) is a minimally invasive treatment for patients with aortic stenosis, where the diseased native valve is replaced by a prosthetic valve delivered via a catheter. Although TAVI has demonstrated clinical success over the past two decades (Angioletti et al. 2024; Cahill et al. 2018), it remains associated with complications such as conduction disturbances, requiring permanent pacemaker implantation and paravalvular leakage (PVL) (Ishizu et al. 2022; Condado et al. 2017; Sherif et al. 2010) (Fig. 1). To address these complications, TAVI devices continue to undergo iterative development. However, the process from novel device design, to regulatory approval and clinical implementation is lengthy and costly. The process includes extensive bench testing, animal studies, and multi-phase clinical trials (Morrison et al. 2018). In addition to their financial and time burdens, animal and clinical studies raise ethical concerns and are often constrained by limited sample sizes.

Integration of in silico trials into the validation chain for regulatory approval offers a promising approach to accelerate this process (Henney et al. 2015; Morrison et al. 2018; Viceconti et al. 2017). These trials enable testing of novel or improved TAVI devices and implantation procedures on large virtual patient cohorts, where individuals with aortic stenosis are represented by computational models. Examples of such computational models include finite element analysis (FEA), computational fluid dynamics (CFD), and fluid–structure interaction (FSI) models, that can be used to simulate the performance of the aortic valve prior, during and after valve replacement. Computational models require patient data, such as aortic valve geometries, as input. However, the use of real patient data is often challenging due to limited availability, incomplete datasets and privacy concerns. To address this challenge, we introduced a virtual cohort generator (VCG) capable of producing synthetic aortic stenosis geometries, in an earlier study (Verstraeten et al. 2024. The VCG can produce virtual cohorts with geometries that are both physiologically plausible and statistically representative of real patient populations. The feasibility of using virtual cohorts with large number of synthetic geometries, for in silico trials has not yet been investigated.

This feasibility can be addressed by conducting a real-world clinical study in silico and compare the model results with the real clinical findings. Previous studies already have demonstrated the potential of patient-specific computational models to simulate TAVI and predict post-procedural outcomes. For instance Grossi et al. (2024) virtually replicated seven real-world TAVI procedures using a FEA model and reported strong agreement between in silico and clinically observed final stent configurations. Similarly, several studies successfully predicted post-TAVI paravalvular leakage (PVL) using patient-specific data in CFD (Bianchi et al. 2019; Mao et al. 2018), and FSI (Luraghi et al. 2019) simulations. Wang et al. Wang et al. (2014) used FEA to simulate TAVI in three patients to predicted aortic root rupture risk and found promising results.

**Fig. 2:**
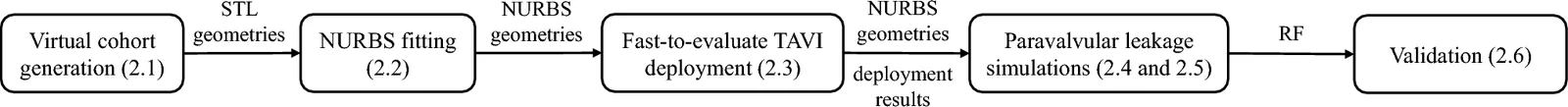
Flowchart summarising the steps to perform in silico trials. The PVL output metric is regurgitant fraction (RF). Corresponding sections are indicated

While these studies highlight the potential of in silico modelling, they are limited by small sample sizes, typically ranging from one to seven patients. In silico trials for device and procedure validation, require model evaluations across large and diverse patient datasets. However, the high computational cost of FEA, CFD, and FSI models, up to seven days per simulation, makes these type of models unsuitable for large-scale studies. Spanjaards et al. (2024) developed a fast in silico model for PVL estimation using a thin-film approximation (Shvarts and Yastrebov 2018), validated against high-fidelity CFD simulations and in vitro experiments. While the leakage model was efficient, the deployment simulations still relied on FEA simulations, limiting scalability. Additionally, their model was tested on only one virtual patient.

To fill this gap, this study aimed to assess the feasibility of conducting large-scale in silico TAVI trials using the VCG by Verstraeten et al. (2024) in combination with a novel fast-to-evaluate model. The model combines a simplified, but fast TAVI deployment model, with a fast 0D lumped parameter model for PVL estimation. Specifically, we will conduct an in silico trial where we investigate anatomical and procedural predictors of PVL and compare our in silico results to the outcomes of the real clinical study of Sherif et al. (2010).

## Methods

Sherif et al. (2010) investigated anatomical and procedural predictors of aortic regurgitation (AR) after implantation of the Medtronic CoreValve bioprosthesis. AR is defined as the retrograde flow of blood (both transvalvular and paravalvular) into the left ventricle (LV). Paravalvular AR specifically refers to the leakage through the space between the aortic root wall and the implanted TAVI device (i.e. PVL) (Fig. 1). Sherif et al. (2010) enrolled 50 patients with severe aortic stenosis who underwent successful TAVI. To enable comparison of our in silico trial to their clinical outcomes, a virtual cohort of aortic stenosis geometries was generated and filtered based on cohort statistics derived from their patient population (Sect. 2.1). The novel fast-to-evaluate deployment model requires input geometries in the form of non-uniform rational B-splines (NURBS), Sect. 2.2 details the algorithm used to fit a NURBS template to the stereolithography (STL) format geometries of the virtual cohort. Subsequently, TAVI deployment simulations were performed using this fast-to-evaluate model, as described in Sect. 2.3. The NURBS geometries and the deployment outcomes (Sect. 2.4) served as input for a 0D lumped-parameter model that estimated regurgitant fraction (RF), that is a metric for PVL, as outlined in Sect. 2.5. Finally, the results were compared to those reported in the clinical study by Sherif et al. (2010) to validate the feasibility to conduct large-scale in silico TAVI trials, using the VCG and the fast-to-evaluate model (Sect. 2.6). A summary of these steps is depicted in Fig. 2.

### Virtual cohort generation

The VCG, introduced by Verstraeten et al. (2024), was extended with several new functionalities to better match the patient population described in the clinical study by Sherif et al. (2010). For full methodological and theoretical details on the VCG, the reader is referred to Verstraeten et al. (2024). First of all, gender-specific cohort generation was enabled by allowing sampling from copula distributions fitted separately to male and female datasets. This addition made it possible to generate male-only or female-only virtual cohorts. Secondly, age was incorporated as an additional variable in the 33-dimensional copula distribution, ensuring that each virtual patient was assigned a realistic age. Finally, the filtering options were extended by adding filters for annulus diameter, sinus diameter, aortic valve area (AVA), and age. These filters were implemented in the same manner as previously done for the angle between the left ventricular outflow tract (LVOT) and the ascending aorta filter (Verstraeten et al. 2024), allowing for more precise control over the anatomical and demographic ranges in the generated cohort.

Using the extended version of the VCG, a virtual cohort was generated to closely resemble the patient population in Sherif et al. (2010), which included 50 patients (40% male) with severe aortic stenosis (AVA < 1 cm$^2$) who successfully underwent TAVI with the Medtronic CoreValve bioprosthesis. To leverage the scalability of the VCG, a larger cohort of 500 virtual patients was created, while maintaining the same male-to-female ratio (200 male, 300 female).

Filtering was applied based on age, AVA, angle, and diameters of the sinus and annulus. To reflect severe aortic stenosis, AVA was constrained to values below 1 cm$^2$, resulting in filtering range 0–1 cm$^2$. Annulus diameter was limited to the range of 20–26 mm, consistent with the use of 26 and 29 mm CoreValve Piazza et al. (2012). The diameter of the CoreValve used is always chosen larger than the annulus diameter, which is referred to as oversizing Lerakis et al. (2013). The remaining parameters were constrained within range $\mu \pm 3\sigma$, based on the means (μ) and standard deviations (σ) reported by Sherif et al. (2010). A summary of the filtering criteria is provided in Table 1.

**Table 1:** Filtering criteria for the virtual cohort of 500 (40% male) patients

	Minimum	Maximum
Annulus diameter (mm)	20	26
Sinus diameter (mm)	20	40
Aortic valve area (cm$^2$)	0	1
Angle (degree)	0	43
Age (years)	57	104

### NURBS fitting

**Fig. 3:**
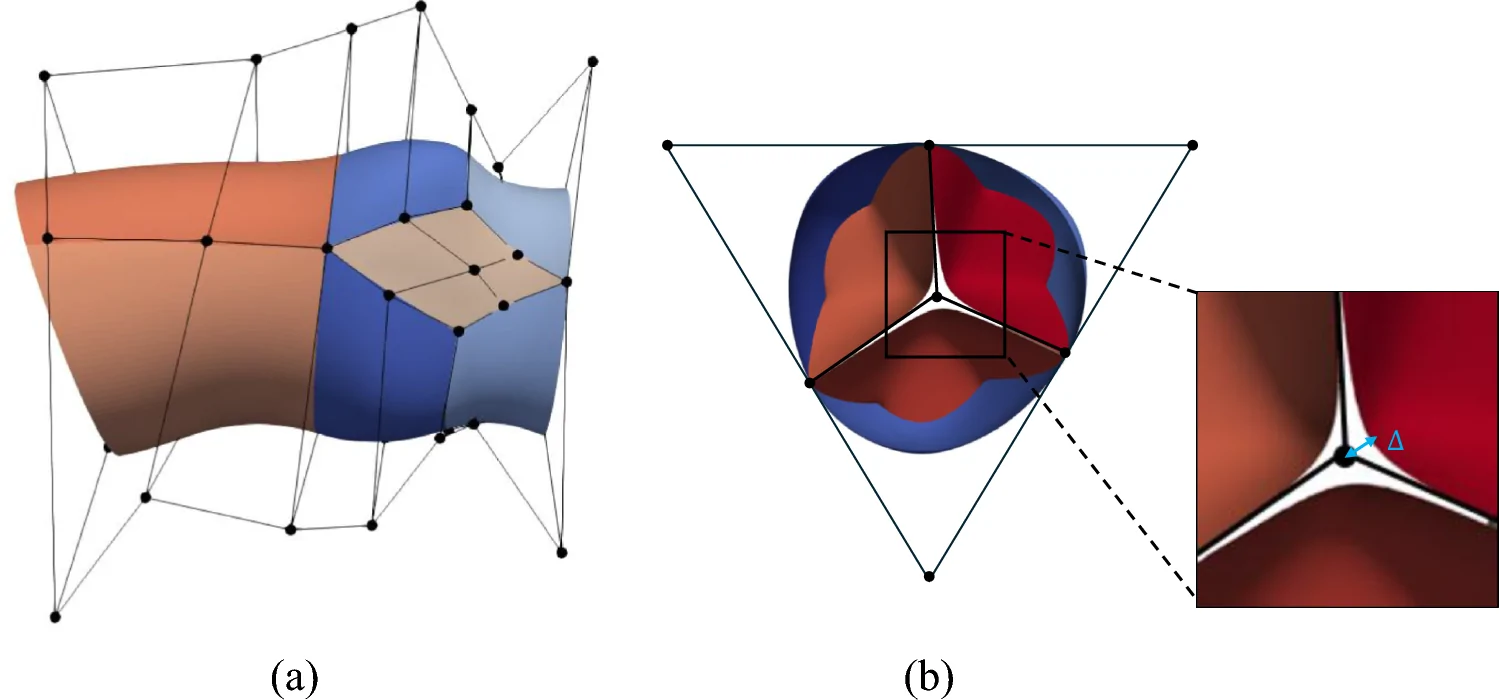
Side view (**a**) and top view (**b**) of the NURBS aortic valve template, showing the control net and control points in black. In (**a**), the aorta, sinus, LVOT, and middle (diamond-shaped) patches are colored orange, blue, light blue, and grey, respectively. In (**b**), leaflet patches are shown in red and sinus patches in blue. The opening parameter Δ is indicated in light blue

The aortic valve stenosis geometries in the virtual cohort were generated in STL format, as this is the native output format of the virtual cohort generator. Although the subsequent fast-to-evaluate model operates on NURBS representations, direct generation of NURBS geometries is not currently supported. Therefore, the generated STL geometries were converted to NURBS using an adapted version of an already available fitting algorithm.

For a comprehensive theoretical background on NURBS we refer to Piegl and Tiller (2012) and Rogers (2000). Unlike the STL meshes that use many small triangles to approximate curves, NURBS can describe smooth, curved geometries, such as the aortic valve, using only a few parameters. NURBS surfaces or curves, are constructed from *n* B-spline basis functions, that are piecewise polynomial curves. These basis functions are controlled by a control net of *n* control points, combined with *n* weights. By adjusting the weights, the surface can be pulled closer to or pushed away from specific control points, allowing for manipulation of the geometry.

To represent the aortic valve, we used a multi-patch NURBS template geometry. The template consisted of 15 interconnected NURBS patches: three aortic, three sinus, three middle, three LVOT, and three leaflet patches. The template is visualised in Fig. 3. Each patch was parametrised by quadratic NURBS resulting in nine control points and weights. The leaflet patches were parametrised by an opening parameter Δ, defined as the distance between the leaflet patch tip and the centerline of the aortic valve geometry. This parameter allowed for control over the leaflet opening state by adjusting the relevant control points and weights, highlighting the advantage of using NURBS. Such direct and efficient control over leaflet opening is not readily achievable with STL-based representations.

To match the template geometry to patient-specific STL geometries, we employed a fitting algorithm adapted from Willems et al. (2024). The algorithm minimises the spatial difference between the template NURBS surface and the target STL point cloud while preserving inter-patch $C^1$-continuity. This continuity requires coinciding control points and aligned surface normals along the different patch interfaces. The fitting process begins with a data point-to-surface projection, assigning each STL vertex to its nearest NURBS patch. This projection enables the computation of displacement error vectors, which iteratively pull the NURBS surface toward the target point cloud. For a more detailed description of the fitting algorithm we refer to Willems et al. (2024). Several modifications were made to the original algorithm to suit our application. First, the proximal and distal edge of the template geometry were constrained to lie within the planes defined by the corresponding edges of the STL geometry. Likewise, the proximal and distal edges of the leaflet patches were constrained to the planes defined by the commissure and hinge points of the target leaflets. Moreover, during data point-surface projection, STL leaflet points were strictly assigned to the corresponding leaflet patches, preventing non-leaflet points to be assigned to leaflet patches. After fitting, the resulting NURBS geometry respresented the valve in its open state, consistent with the STL target geometry. However, the deployment algorithm required a closed initial configuration. To achieve this configuration, the leaflet patches were closed by adjusting the opening parameter Δ, effectively repositioning the leaflet tips toward the valve centerline.

### Fast-to-evaluate TAVI deployment simulations

**Fig. 4:**
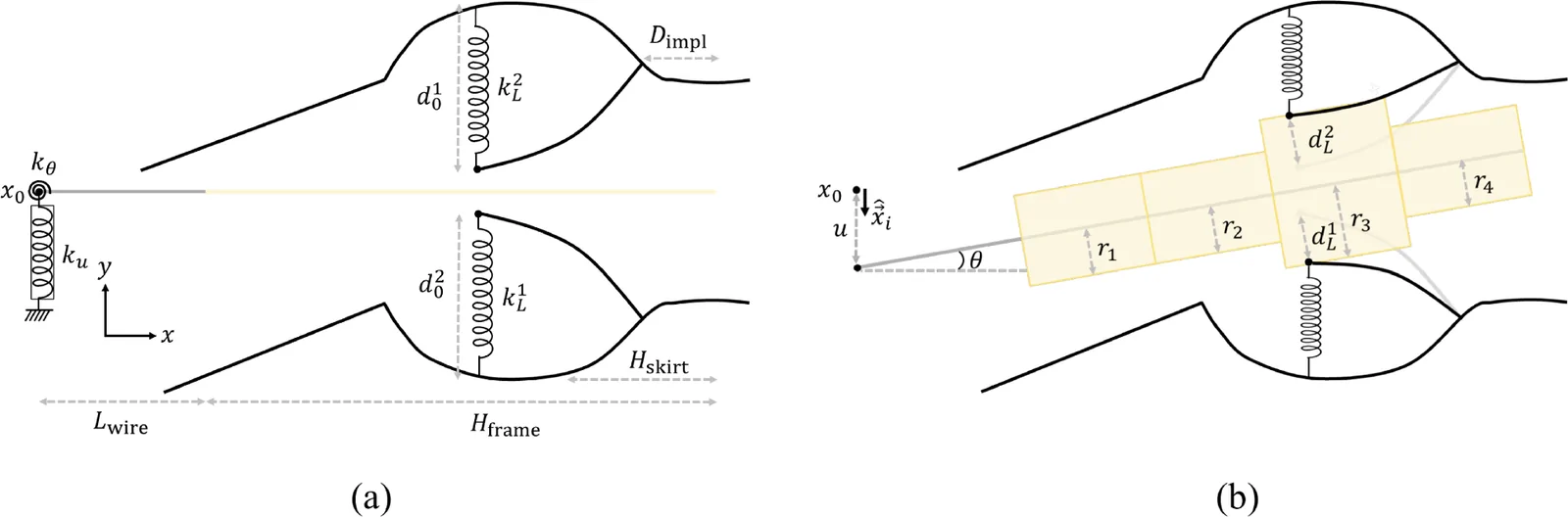
Schematic representation of the TAVI deployment model at two stages: **a** initial configuration and **b** intermediate configuration. Relevant model parameters are indicated, with spring constants *k* and cylinder expansion rates $\dot{r}$

#### Mechanical model

For the in silico TAVI trials, we developed a fast-to-evaluate TAVI deployment model. The model simulates the clinical TAVI procedure with a prosthetic self-expanding valve (SEV), like the Medtronic CoreValve used in the study of Sherif et al. (2010). During the clinical procedure, a guiding catheter moves the self-expanding valve to the stenotic aortic valve, where the valve expands and displaces the native leaflets until it is in contact with the aortic root wall. The prosthetic SEV consist of a frame partially covered by a “skirt” that blocks blood flow (Fig. 1). In the fast-to-evaluate model, a few simplifications and assumptions were made. The SEV was modelled as an expanding cylinder that adapts to the surrounding anatomy via applied forces. The exact frame geometry was omitted, as the focus was on predicting PVL. For the same reason, the prosthetic leaflets were not modelled, and the aortic root was assumed rigid.

Figure 4a illustrates a 2D schematic of the initial configuration of the deployment model. A guide wire was positioned within the aorta, extending beyond the annulus by a distance $D_{\text {impl}}$, representing the implantation depth. The expanding cylinder with height $H_{\text {frame}}$, was subdivided into four equal segments (each of length $\frac{H_{\text {frame}}}{4}$) with radius $r_{s}$, with s∈[1,2,3,4] (Fig. 4b). Initially, all segments had a small radius (0.5 mm), mimicking the crimped state of the SEV. The guide wire could translate and rotate freely around a reference point $x_0(u,v)$, with *u* and *v* denoting translations in the *y*- and *z*-directions, and θ and ϕ representing rotations around the *z*- and *y*-axes, respectively. The distance from the rotation point to the first cylinder segment was $L_{\text {wire}}$.

During the expansion of the cylinder, three types of spring-based forces were considered. The first type modelled the contact between the expanding cylinder and the native leaflets. Each leaflet was modelled as a spring that connected its leaflet tip to the sinus. The springs applied a force to the guide wire when they were compressed by the expanding cylinder:

1 $$F_{L} = k_{L} d_{L} ,$$

with $d_L$ the compression, defined as the distance between the initial leaflet tip position and the expanding cylinder (Fig. 4b), and $k_L$ the corresponding spring stiffness.

The second type of forces modelled the contact between the expanding cylinder and the aortic root wall. The contact was modelled at a set of $n_w$ points, divided over the wall. The distance between a wall point and the expanding cylinder was defined as

2 $$\begin{aligned} d_w = r_{s}(t) - d_g(t), \end{aligned}$$

with $d_g$ the distance between the wall point and the closest point on the guide wire. When the distance $d_w$ was positive, i.e. when the cylinder radius exceeded the local aortic root radius, the wall point was considered to be in contact with the cylinder, and a repelling force was applied to the guide wire according to

3 $$\begin{aligned} F_{\text {w}} = k_wd_w, \end{aligned}$$

with spring stiffness $k_w$.

The last type of force involved the internal resistance at the origin of the guide wire that ensured a controlled response of the guide wire’s position to the external forces applied by the wall and leaflets. The origin contained two translational springs with stiffnesses $k_u$ in the *y*-direction and $k_v$ in the *z*-direction. Additionally, it contained two rotational springs with stiffnesses $k_{\theta }$ around the *z*-axis and $k_{\phi }$ around the y-axis.

Inertial forces were assumed to be negligible, resulting in the quasi-static equilibrium described by the following non-linear system of equations:

4 $$\begin{aligned} \begin{aligned}&k_u(u-u_0) + \sum _{i=1}^{n} \vec {F}_i(u,v,\theta ,\phi )\cdot \vec {e}_y = 0, \\&k_v(v-v_0) + \sum _{i=1}^{n} \vec {F}_i(u,v,\theta ,\phi )\cdot \vec {e}_z = 0, \\&k_\theta (\theta -\theta _0) + \sum _{i=1}^{n} \vec {M}_i(u,v,\theta ,\phi )\cdot \vec {e}_y = 0, \\&k_\phi (\phi -\phi _0) + \sum _{i=1}^{n} \vec {M}_i(u,v,\theta ,\phi )\cdot \vec {e}_z = 0, \\ \end{aligned} \end{aligned}$$

with *n* the total number points ($n_w$ wall points and three leaflet points). The moments were defined as $\vec {M}_i(u,v,\theta ,\phi )=\hat{\vec {x}}_i(u,v,\theta ,\phi ) \times \vec {F}_i(u,v,\theta ,\phi )$, with $\hat{\vec {x}}_i$ the unit vector from the reference point $x_o$ to the contact point on the guide wire.

#### TAVI deployment algorithm

During the TAVI deployment simulation, the cylinder segments were iteratively expanded. In each iteration *i*, the radius of each cylinder segment *s* was updated according to:

5 $$\begin{aligned} r_{s,i} = r_{s,i-1} + \Delta t\ \cdot \dot{r}_s, \end{aligned}$$

where Δt is the step size and $\dot{r}_s$ is the predefined expansion rate of the segment (Fig. 4b). Each cylinder segment had its own initial expansion rate that was dependent on $\dot{r}_1$ via correlation parameters α and β as follows:

6 $$\begin{aligned} \mathop {{\dot{r}}}\limits _{\sim } = \begin{bmatrix} \dot{r}_1 \\ \dot{r}_2\\ \dot{r}_3 \\ \dot{r}_4 \end{bmatrix} = \begin{bmatrix} \dot{r}_1 \\ \alpha (\dot{r}_{1,\text {max}} - \dot{r}_1) + \dot{r}_1 \\ \dot{r}_2 + \beta \\ \dot{r}_3 + \alpha \end{bmatrix}, \end{aligned}$$

with $\dot{r}_{1,{\text {max}}}$ the upper limit for the expansion rate of the first segment. Correlation parameters α and β were used to ensure that the expansion rate of the two proximal cylinder segments were higher, compared to the two distal segments. This difference was consistent with what was observed in the clinic and led to more realistic stent shapes after visual inspection of initial tests. Following the radius update, the model computed the distances between the cylinder and the aortic wall $d_w$, and the leaflet tips $d_L$. These distances were used to calculate the corresponding spring forces. The spring forces influenced the position and orientation of the guide wire, defined by the variables $u, v, \theta , \phi$, that were obtained by solving the system of equations defined in Eq. 4. Initially, the segment expansion rates $\dot{r}_s$ were kept constant, allowing for relatively large steps. If predefined criteria were violated, indicating that the step size was too large, the expansion rate of the affected segment was reduced (halved) and the step was repeated. This adaptive procedure was continued until convergence was reached. The criteria included:

- The cylinder segment exceeded the aortic wall points by more than a predefined maximum oversizing parameter.
- Leaflet spring forces exceeded a maximum allowable value $F_{\text {L,max}}$, which was derived from input parameter $\hat{d}_{\text {post}}$, that is the estimated post deployment distance between the leaflet tips and the sinus wall:
  7 $$\begin{aligned} F_{\text {L,max}} = k_L\left( \left( \frac{1}{3}\sum _{j=1}^3d_0^j\right) - \hat{d}_{\text {post}} \right) , \end{aligned}$$
  where $d_0^j$ is the initial distance between leaflet tip *j* and the sinus wall (Fig. 4a). In other words, a smaller value for $\hat{d}_{\text {post}}$ allows for a higher leaflet spring compression, and thus a higher maximum force.

The iterative expansion algorithm continued until either all segment expansion rates fell below a predefined tolerance or a maximum of 1000 iterations was reached.

To ensure realistic deployment, two constraints were incorporated into the deployment algorithm. the leaflet tips were kept within segment 3 throughout the simulation. Since the leaflet springs in segment 3 were stiffer than the wall springs in the other segments, expansion was more constrained in this region, resulting in a smaller local stent diameter and contributing to the characteristic hourglass shape of the deployed stent. To ensure that the leaflet tips remained within segment 3 independent of the patient-specific geometry, the segment boundaries were adjusted when necessary. Specifically, if a leaflet tip was located within 5% of the segment length from a segment boundary, the boundary was shifted by 5% to keep the leaflet tip within segment 3 throughout the simulation. This also prevented abrupt changes in local expansion behaviour during deployment. Second, the algorithm monitored whether the guide wire moved beyond the initial position of the leaflet tips, i.e. when the guide wire line in Fig. 4a was no longer in between the two black dots, a scenario that is anatomically implausible. If this scenario occurred, the expansion rates of all segments were halved and the step was repeated. If the issue persisted over several iterations, the simulation was terminated to avoid non-physiological results.

After each iteration in the algorithm, the guide wire’s position and orientation were reconstructed using the updated simulation outputs u,v,θ and ϕ. The stent configuration was estimated by smoothing the cylinder segments with radius $r_s$, providing a continuous representation of the deployed stent. For each leaflet, the opening parameter Δ was derived from the current spring compression $d_L$. While these reconstructions could be performed at every iteration for visualisation purposes, only the final configuration was used for PVL estimation.

### Leakage gap reconstruction

**Fig. 5:**
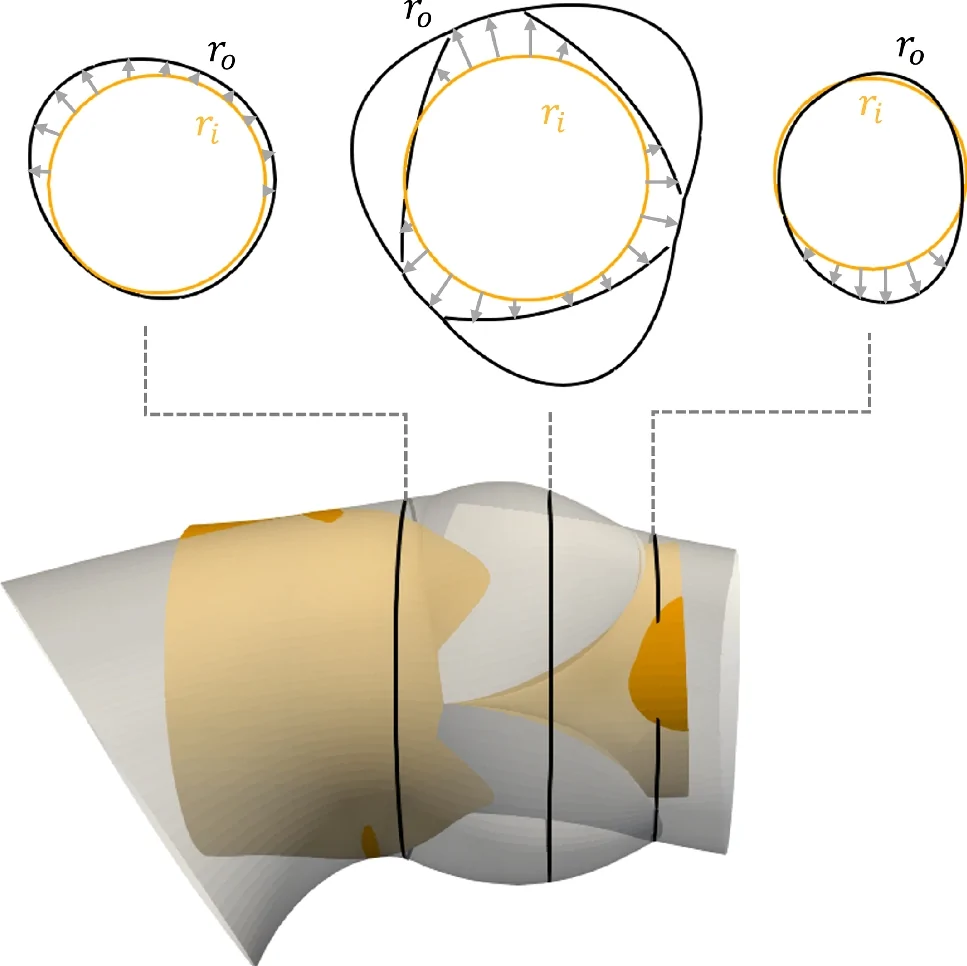
Visualisation of the leakage gap at three different slices throughout the aortic valve geometry, with $r_i$ and $r_o$ the radial distances from the guide wire to the cylinder and the aortic wall, respectively. The aortic valve geometry is in white, and the cylinder in orange. The gap heights are indicated with the grey arrows

To estimate PVL, the geometry of the gap between the final deployed stent and the native aortic valve was reconstructed. The stent cylinder was discretised into 100 axial slices, perpendicular to the guide wire, along its total length $H_{\text {frame}}$. Each slice was further divided into 100 angular segments, resulting in a total of $1\cdot 10^4$ volumetric cells. For each cell, the gap height *h* was calculated as the difference between the radial distance from the guide wire to the aortic root wall ($r_o$) and the radial distance from the guide wire to the cylinder surface ($r_i$): $h = r_o - r_i.$ If the cylinder surface exceeded the aortic wall ($r_i > r_o$) in a certain cell, the gap height was set to zero, so no leakage was possible through that cell. These cells were excluded from the final leakage gap geometry. The leakage gap is visualised for three different slices in Fig. 5. PVL occurs only along the skirt region of the stent, as blood can flow through the stent frame but not through the skirt material. Therefore, only the most proximal slices, up to including the slice corresponding to the skirt height, were considered in the PVL estimation. We assumed that in the skirt region, the slices were approximately perpendicular to the blood flow direction, yielding a reliable estimation of the leakage gap.

### Lumped parameter leakage model

**Fig. 6:**
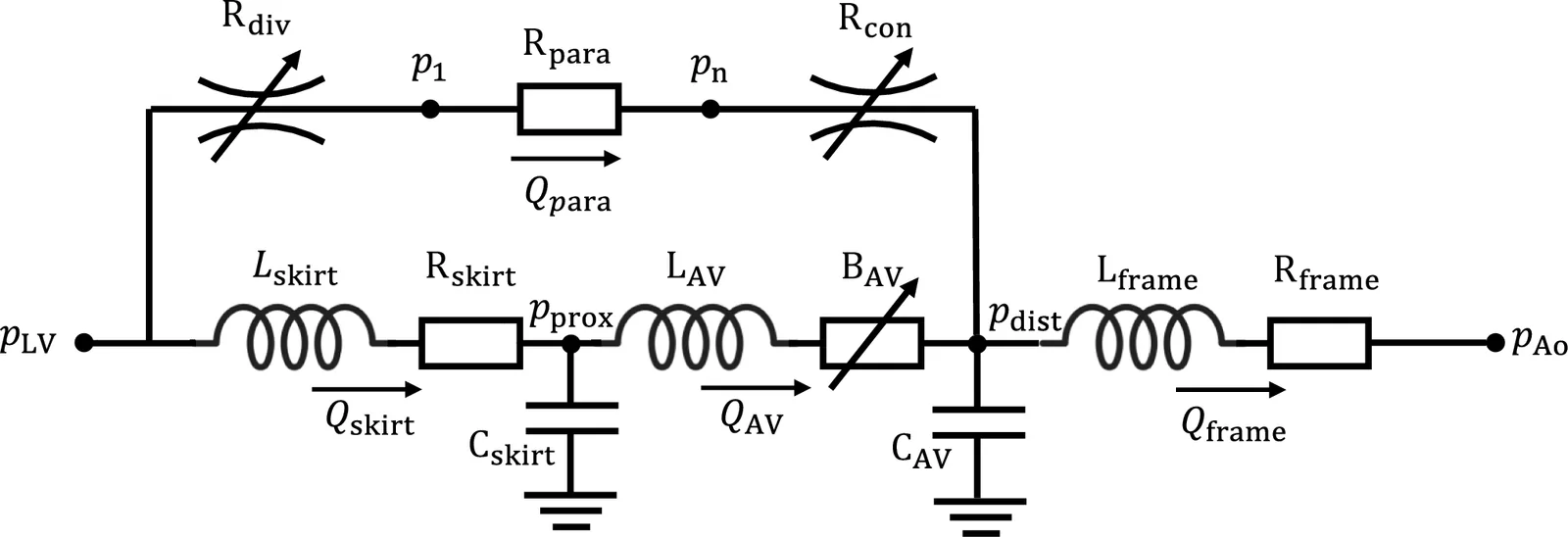
Schematic 0D lumped parameter leakage model with skirt, valve (AV), frame and paravalvular (para) segments, and *L*, *R*, *B*, *C*, *p*, and *Q* representing inductances, resistors, Bernoulli resistors, capacitors, pressures and flows, respectively. Note that the aorta is depicted on the right, in contrast to previous figures

PVL was estimated using a 0D lumped parameter model that is visualised in Fig. 6. The model consisted of four distinct segments: paravalvular, skirt, frame, and valve segments, each addressed individually below.

#### Paravalvular segment

During the diastolic phase of the cardiac cycle, when the prosthetic valve is closed, blood may leak back through the gap between the deployed stent and the aortic wall (PVL). This paravalvular flow was modelled as laminar flow through an annular space between two concentric cylinders, assuming no-slip boundary conditions at both the inner and outer surfaces. As patients are in a supine position during the procedure, gravitational effects on the flow were considered negligible and therefore omitted. Following the formulation described by White (2011) the pressure drop across the leakage gap was defined as:

8 $$\begin{aligned} p_1 - p_n = Q_{\text {para}}R_{\text {para}}, \end{aligned}$$

with $p_1$ and $p_n$ the pressures in the first and last slices of the stent’s skirt, respectively, $Q_{\text {para}}$ the paravalvular flow rate, and $R_{\text {para}}$ the effective resistance of the leakage gap along the skirt slices. The effective resistance was computed as the sum of the individual resistances of the *n* skirt slices (White 2011):

9 $$\begin{aligned} R_{\text {para}} = \sum _{s=1}^n \frac{16\mu }{\Theta _s}\left( r^4_{o,s} - \frac{\left( r^2_{o,s} - r^2_{i,s} \right) ^2}{\text {ln}\left( \frac{r_{o,s}}{r_{i,s}}\right) } - r^4_{i,s} \right) ^{-1}\Delta s, \end{aligned}$$

with $r_{o,s}$ and $r_{i,s}$, the average inner and outer radii of slice *s*, respectively, μ the dynamic viscosity of blood, $\Theta _s$ the angular portion of the slice (between 0 and 2π) where the gap height is non-zero, and Δs the slice thickness.

In addition to the pressure drop due to the leakage gap resistance, two extra pressure losses were considered:

- Pressure loss due to rapid converging flow from the aorta into the narrow leakage gap.
- Pressure loss due to rapid diverging flow from the narrow leakage gap into the LVOT.

These pressure losses were modelled as minor losses using the Bernoulli equation with loss coefficients, as described by White (2011):

10 $$\begin{aligned} \frac{p_1}{\rho g} + \alpha \frac{v_1^2}{2g} + z_1 = \frac{p_2}{\rho g} + \alpha \frac{v_2^2}{2g} + z_2 + \frac{v_1^2}{2g}K + \frac{v_2^2}{2g}K, \end{aligned}$$

with pressure *p*, velocity *v*, height *z*, density ρ, gravitational constant *g*, and kinetic energy correction factor α, which equals 2 for laminar flow. Subscripts 1 and 2 denote the locations upstream and downstream, the converging/diverging flows, respectively. The loss coefficients for the rapid converging and diverging flows were defined as (White 2011):

11 $$\begin{aligned} K_{\text {con}}&= \lambda \left( 1 - \frac{a}{A} \right) , \end{aligned}$$

12 $$\begin{aligned} K_{\text {div}}&= \left( 1 - \frac{a}{A} \right) ^2, \end{aligned}$$

respectively, with *a* the smaller cross-sectional area, *A* the larger one and λ a constant with value 0.42 (-). By assuming supine patients ($z_1 = z_2$), and substituting $v = \frac{Q_{\text {para}}}{A}$ in Eq. 10, the pressure drops due to rapid converging and diverging flows are:

13 $$\begin{aligned}&p_{\text {dist}} - p_n = R_\text {con}Q^2_{\text {par}} \\ & \quad = \left( \frac{\rho }{2A_{\text {dist}}^2}\left( K_{\text {con}} - \alpha \right) + \frac{\rho }{2A_n^2}\left( K_{\text {con}} + \alpha \right) \right) Q_{\text {para}}^2 \end{aligned}$$

14 $$\begin{aligned}&p_1 - p_{\text {LV}} = R_{\text {div}}Q_{\text {para}}^2\\ & \quad= \left( \frac{\rho }{2A_1^2}\left( K_{\text {div}} - \alpha \right) + \frac{\rho }{2A_{\text {LV}}^2}\left( K_{\text {div}} + \alpha \right) \right) Q_{\text {para}}^2 \end{aligned}$$

with $p_{\text {LV}}$ and $p_{\text {dist}}$, the pressures in the LV and distal to the valve, respectively, with corresponding cross-sectional areas $A_{\text {LV}}$ and $A_{\text {dist}}$. Cross-sectional gap areas in the first and last skirt slices were denoted with $A_1$ and $A_n$, respectively.

When the valve is open, the majority of blood passes through the prosthetic valve, but a small fraction may still flow through the leakage gap. However, during forward flow, the pressure losses due to the rapid converging and diverging flows were assumed to be negligible and therefore omitted. Combining Eqs. 8, 13, and 14 the total pressure drop across the complete paravalvular segment equals

15 $$\begin{aligned}&p_{\text {LV}} - p_{\text {dist}} \\& \quad= {\left\{ \begin{array}{ll} R_{\text {par}}Q_{\text {par}} & \text {if } Q_{\text {par}} > 0 \\ R_{\text {par}}Q_{\text {par}} + R_{\text {con}}Q_{\text {par}}^2 + R_{\text {div}}Q_{\text {par}}^2 & \text {if } Q_{\text {par}} < 0 \end{array}\right. }. \end{aligned}$$

In summary, when the prosthetic valve is open ($Q_{\text {para}} > 0$ mL/s), the leakage flow is governed solely by the gap resistance. When the prosthetic valve is closed ($Q_{\text {para}} < 0$ mL/s), additional pressure losses due to converging and diverging flows were included.

#### Skirt and frame segments

The skirt and frame segments both consisted of a viscous tube resistance (*R*) and an inductance (*L*) in series. The skirt segment represents the flow through the tube defined by the expanded cylinder in the skirt slices (radius $r_i$), while the frame segment represents the flow through tube defined by the aortic root wall (radius $r_o$), since the blood cannot flow through the skirt but can flow through the frame. The pressure drops across the skirt and frame segments were given by:

16 $$\begin{aligned} \Delta p_{\text {skirt}}&= R_{\text {skirt}}Q_{\text {skirt}} + L_{\text {skirt}}\frac{dQ_{\text {skirt}}}{dt}, \end{aligned}$$

17 $$\begin{aligned} \Delta p_{\text {frame}}&= R_{\text {frame}}Q_{\text {frame}} + L_{\text {frame}}\frac{dQ_{\text {frame}}}{dt}, \end{aligned}$$

with $Q_{\text {skirt}}$ and $Q_{\text {frame}}$ the flows through the skirt and frame segments, respectively. The resistances and inductances were calculated using

18 $$\begin{aligned} R = \frac{8\mu l}{\pi r^4}, \end{aligned}$$

and

19 $$\begin{aligned} L = \frac{\rho l}{\pi r^2}, \end{aligned}$$

where *l* is the length of the corresponding segment and *r* the average radius along the segment. The length of the skirt segment equalled $H_{\text {skirt}}$, and the valve segment was assumed to have the same length. The frame segment modelled all remaining slices not part of the skirt or valve segments, resulting in a length of $H_{\text {frame}} - 2H_{\text {skirt}}$. Note that while the full frame height of the Medtronic CoreValve is $H_{\text {frame}}$, the frame segment in the lumped parameter model, only includes the slices outside the skirt and valve segments. The nodal pressures proximal and distal to the valve were computed from the corresponding volumes and compliances:

20 $$\begin{aligned} p_{\text {prox}}&= \frac{V_{\text {prox}}}{C_{\text {skirt}}}, \end{aligned}$$

21 $$\begin{aligned} p_{\text {dist}}&= \frac{V_{\text {dist}}}{C_{\text {AV}}}, \end{aligned}$$

with $V_{\text {prox}}$ and $V_{\text {dist}}$ the nodal volumes and $C_{\text {skirt}}$ and $C_{\text {AV}}$ the respective compliances of the skirt and the valve segments. The compliances were assumed to be equal to that of the ascending aorta and were based on values found by Tobey et al. (2019). The volume changes over time were approximated using backward differencing:

22 $$\begin{aligned} \frac{dV_{\text {prox}}}{dt}&\approx \frac{V_{\text {prox,n}} - V_{\text {prox,n-1}} }{\Delta t} = Q_{\text {skirt}} - Q_{\text {AV}},\end{aligned}$$

23 $$\begin{aligned} \frac{dV_{\text {dist}}}{dt}&\approx \frac{V_{\text {dist,n}} - V_{\text {dist,n-1}} }{\Delta t} = Q_{\text {AV}} + Q_{\text {para}}- Q_{\text {frame}}, \end{aligned}$$

with time step size Δt, and subscripts *n* and *n*-1 indicating the current and previous time step, respectively. Combining Eqs. 16–23 yields Equations for flow through the skirt and frame segments:

24 $$\begin{aligned} \left( R_{\text {skirt}}+ \frac{\Delta t}{C_{\text {AV}}}\right) Q_{\text {skirt}} + L_{\text {skirt}}\frac{dQ_{\text {skirt}}}{dt} \\\quad= p_{\text {LV}} + \frac{1}{C_{\text {AV}}}\left( Q_{\text {AV}}\Delta t + V_{\text {prox,n-1}} \right) \end{aligned}$$

25 $$\begin{aligned} \left( R_{\text {frame}}+ \frac{\Delta t}{C_{\text {skirt}}}\right) Q_{\text {frame}} + L_{\text {frame}}\frac{dQ_{\text {frame}}}{dt} \\ \quad= \frac{1}{C_{\text {skirt}}}\left( \left( Q_{\text {para}} + Q_{\text {AV}} \right) \Delta t + V_{\text {dist,n-1}} \right) - p_{\text {Ao}}. \end{aligned}$$

#### Valve segment

The prosthetic valve was modelled using the approach by Mynard et al. (2012). The transvalvular pressure drop was given by:

26 $$\begin{aligned} \Delta p_{\text {AV}} = B_{\text {AV}}Q_{\text {AV}}\left| Q_{\text {AV}} \right| + L_{\text {AV}}\frac{dQ_{\text {AV}}}{dt}, \end{aligned}$$

with $Q_{\text {AV}}$ the transvalvular flow rate, $B_{\text {AV}}$, the Bernoulli resistance and $L_{\text {AV}}$ the inertance of the valve segment. These parameters were defined as:

27 $$\begin{aligned} B_{\text {AV}}&= \frac{\rho }{2\zeta ^2 A_{\text {AV}}^2}, \end{aligned}$$

28 $$\begin{aligned} L_{\text {AV}}&= \frac{\rho l_{\text {AV}}}{A_{\text {AV}}}, \end{aligned}$$

with ρ the blood density, $A_{\text {AV}}$, the effective valve area when fully open, and $l_{\text {AV}}$ the valve segment length. The valve length $l_{\text {AV}}$ was assumed equal to the skirt height $H_{\text {skirt}}$. The effective area $A_{\text {AV}}$ was calculated as:

29 $$\begin{aligned} A_{\text {AV}} = \gamma \pi r^2, \end{aligned}$$

where *r* is the average radius of the valve segment. Since a fully opened valve does not form a perfect circular area due to the presence of leaflets, a correction factor γ = 0.7 was applied, based on findings by Borowski et al. (2018). The valve opening state ζ (ranging from 0 for fully closed to 1 for fully open) depended on the pressure drop across the valve as follows:

30 $$\begin{aligned} \frac{d\zeta }{dt} = {\left\{ \begin{array}{ll} \left( 1 - \zeta \right) K_{\text {vo}} \Delta p_{\text {AV}} & \text {if } \Delta p_{\text {AV}} > 0 \\ \zeta K_{\text {vc}}\Delta p_{\text {AV}} & \text {if } \Delta p_{\text {AV}} < 0 \end{array}\right. }, \end{aligned}$$

with $K_{\text {vo}}$, and $K_{\text {vc}}$, the valve opening and closing rate coefficients, respectively. To compute ζ at each time step *t*, implicit time integration using backward differencing was applied:

31 $$\begin{aligned} \zeta _t = {\left\{ \begin{array}{ll} \dfrac{K_{\text {vo}}\Delta p_{\text {AV}} \Delta t + \zeta _{t-1}}{1+K_{\text {vo}}\Delta p_{\text {AV}} \Delta t} & \text {if } \Delta p_{\text {AV}} > 0 \\ \dfrac{\zeta _{t-1}}{1 - K_{\text {vc}}\Delta p_{\text {AV}} \Delta t} & \text {if } \Delta p_{\text {AV}} < 0 \end{array}\right. }, \end{aligned}$$

with Δt, the time step size. Both, $K_{\text {vo}}$ and $K_{\text {vc}}$ were set to 0.12 Pa$^{-1}$s$^{-1}$, consistent with Mynard et al. (2012).

#### Numerical implementation

**Fig. 7:**
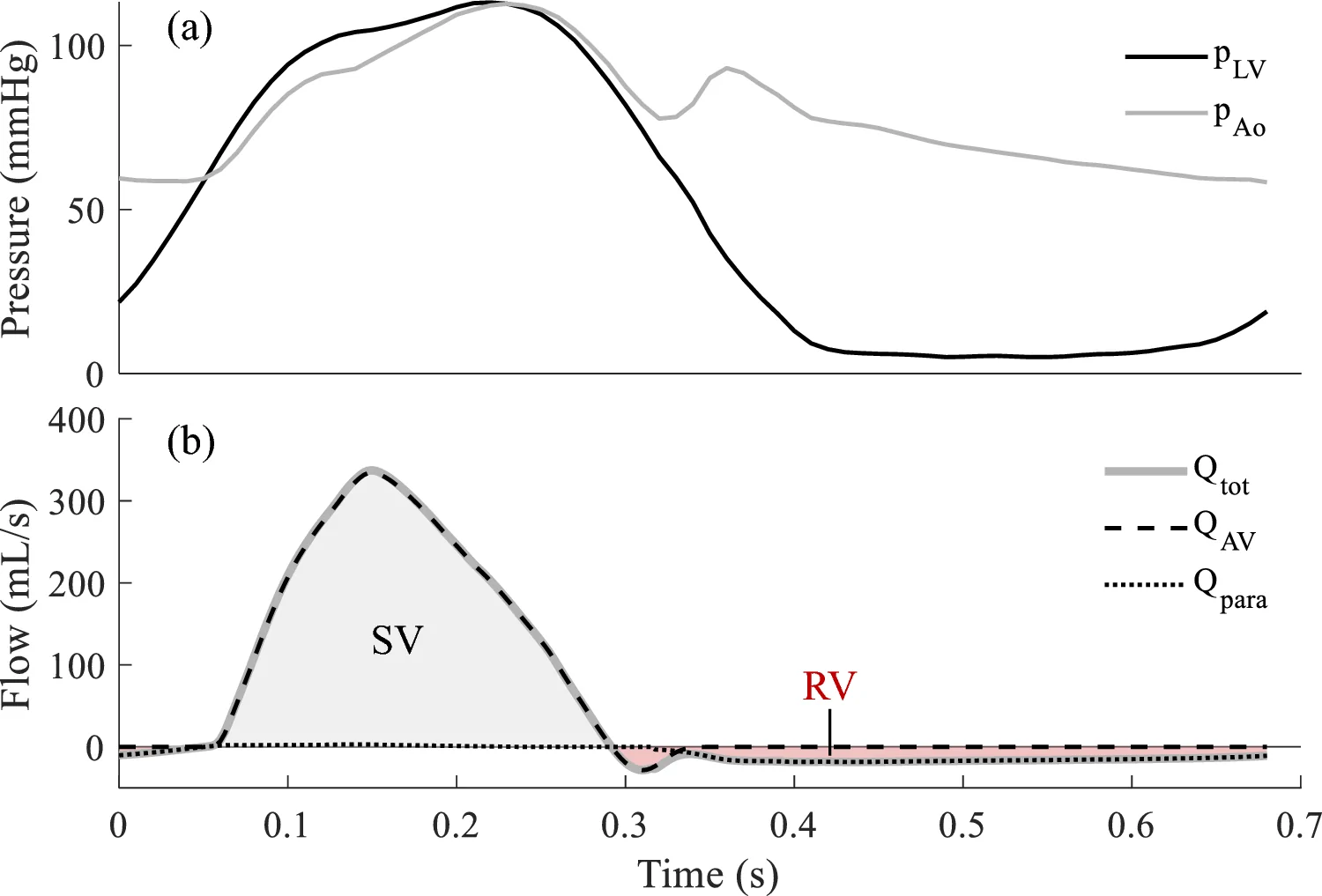
**a** Prescribed left ventricular $p_{\text {LV}}$ and aortic $p_{\text {Ao}}$ pressure waveforms. **b** Total output flow ($Q_{\text {tot}}$), comprising transvalvular flow ($Q_{\text {AV}}$) and paravalvular flow ($Q_{\text {para}}$). Stroke volume (SV) is highlighted in grey, regurgitant volume (RV) in red

The simulation was initialised with the valve in a closed state. Left ventricular ($p_{\text {LV}}$) and aortic ($p_{\text {Ao}}$) pressures were prescribed as input (Sect. 2.6, Fig. 7). Pressures $p_{\text {prox}}$ and $p_{\text {dist}}$ were initially set equal to $p_{\text {LV}}$ and $p_{\text {Ao}}$, respectively. At each time step, the following sequence was executed:

- Transvalvular flow ($Q_{\text {AV}}$) was computed using Eqs. 26–31, applying explicit, implicit and combined time integration schemes. The scheme yielding the lowest error was selected.
- Paravalvular flow ($Q_{\text {para}}$) was calculated using Eq. 15.
- Skirt ($Q_{\text {skirt}}$) and frame segment flows ($Q_{\text {frame}}$) were computed using Eqs. 24 and 25, again testing multiple integration schemes.
- Pressures $p_{\text {prox}}$ and $p_{\text {dist}}$ were updated using Eqs. 20 and 21, respectively.
- The updated opening state $\zeta _t$ was calculated using Eq. 31.

This process was repeated for each time step. The outputs of the lumped parameter leakage model were the transvalvular and paravalvular flows over time.

### In silico trial and validation

The virtual cohort of 500 synthetic patients generated in Sect. 2.1 was used to conduct our in silico trial. In the clinical study of Sherif et al. (2010), patients underwent TAVI using the Medtronic CoreValve. Valve sizing was based on annulus diameter: a 26 mm CoreValve was used for annulus diameters between 20–23 mm, and a 29 mm CoreValve for diameters between 23–26 mm (Piazza et al. 2012), leading to an oversizing of 3 to 6 mm. Accordingly, the frame height $H_{\text {frame}}$ was set to 53 mm for the 26 mm valve and 55 mm for the 29 mm valve (Sherif et al. 2010). The skirt height $H_{\text {skirt}}$ was fixed at 12 mm for all patients, consistent with the CoreValve design (Sinning et al. 2012).

Wall and leaflet stiffnesses, $k_w$ and $k_L$, were kept constant across the cohort. A sensitivity analysis showed that deployment outcomes were insensitive to variations in $k_w$ within the range 1–10$^3$ N/mm, so $k_w$ was fixed at 1 N/mm. Leaflet stiffness $k_L$ was estimated using a high-fidelity solid-shell contact model in Ansys Mechanical (explicit solver), as performed by Divi and Verhoosel (2023), resulting in a mean value of $k_L = 2.8 \cdot 10^{-3}$ N/mm.

To account for the slight expansion of the aortic wall observed in vivo due to stent oversizing (Lerakis et al. 2013), an effect that is not modelled, a maximum oversizing parameter of 3 mm was used for the stopping criterion in the deployment simulations (Sect. 2.3). This value yielded a range of PVL values that was consistent with the range observed clinically (Crouch et al. (2015)) (Sect. 3).

The remaining model parameters $L_{\text {wire}}$, $D_{\text {impl}}$, $\hat{d}_{\text {post}}$, $\dot{r}_1$, α, and β could not be derived on a patient-specific basis. Therefore, multiple deployment and leakage simulations were performed per patient with varying combinations of these six model parameters. Parameter sampling was done using Sobol quasirandom sampling (Bratley and Fox 1988) in Matlab 2021a to ensure proper coverage of the parameter space. Ranges for clinically interpretable parameters, the implantation depth $D_{\text {impl}}$, and the estimated post deployment distance between the leaflet tips and the sinus wall $\hat{d}_{\text {post}}$ were derived from literature, while the remaining ranges were assumptions (Table 2).

To obtain 50 successful simulations per patient, 1.25 × 50 simulations were initially performed. Simulations resulting in errors or outliers were discarded. Outliers in the deployment results were identified using the interquartile range (IQR), and the 25th ($Q_1$) and 75th ($Q_3$) percentiles of the output parameters. Specifically, a simulation was flagged as an outlier if any of the cylinder radii $r_1, r_2, r_3$ or $r_4$ fell below $Q_1-1.5\cdot$IQR, or if any of values for u,v,θ or ψ exceeded $Q_3+1.5\cdot$IQR. These limits were chosen as visual inspection confirmed that cases beyond these thresholds were clearly unphysical. Only non-outlier deployment results were used for the subsequent PVL simulations. This approach yielded at least 50 valid simulations for most patients, although for a few patients fewer than 50 remained after filtering.

**Table 2:** Model input parameter ranges with length of guide wire $L_{\text {wire}}$, implantation depth $D_{\text {impl}}$, estimated post deployment distance between leaflet tips and sinus wall $\hat{d}_{\text {post}}$, initial expansion rate of the first segment $\dot{R}_1$, and expansion rate ratios α, and β (Eq. 6)

Parameter	Minimum	Maximum
$L_{\text {wire}}$ (mm)	1	9
$D_{\text {impl}}$ (mm)^a^	3	6
$\hat{d}_{\text {post}}$ (mm)^b^	0.05	6.2
$\dot{r}_1$ (mm/s)	8	10
α (–)	−0.5	1
β (–)	0	3

$^a$Based on Yudi et al. (2018) and Hafiz and Pinto (2016), $^b$Based on Jabbour et al. (2018) and Abdelghani et al. (2020)

The lumped parameter leakage model required post-TAVI LV and aortic pressure signals. We used post-TAVI signals from a typical patient from the data set of Johnson et al. (2018) (Fig. 7a). From the leakage simulations, transvalvular and paravalvular flow rate signals were extracted to compute regurgitant volume (RV), stroke volume (SV), and regurgitant fraction (RF). RV and SV were calculated by integrating the total flow (sum of transvalvular and paravalvular flow rates) over the backward and forward flow periods, respectively (Fig. 7b). RF was defined as the ratio of RV to SV.

To enable a direct comparison with the results reported by Sherif et al. (2010), we adopted the same grading scheme for AR. Sherif et al. (2010) used a 4-class angiographic grading scheme, categorising PVL as mild (grade I), moderate (grade II), moderate-to-severe (grade III), and severe (grade IV). In their analysis, patients were split based on severity of AR: those with AR ≥ grade II, and those with AR < grade II. According to Pibarot et al. (2015), AR of grade II or higher corresponds to an RF of at least 30%, in a 4-class angiographic grading scheme. Therefore, we classified our simulation results into two groups: RF ≥ 30% (≥ grade II) and AR < 30% (< grade II). We then compared several anatomical and procedural features, such as the angle between the LVOT and the ascending aorta, and the implantation depth, between these two groups, and evaluated whether our findings aligned with those reported in the clinical study (Sherif et al. 2010). In addition to feature distributions, we investigated correlations between PVL and both model parameters and anatomical shape features to assess whether similar relationships were observed as in the clinical study.

### Comparison with high-fidelity model

The results of the fast-to-evaluate model were also compared with the high-fidelity modelling framework of Spanjaards et al. (2024). In their study, stent deployment of a self-expandable CoreValve Evolut was simulated using a high-fidelity explicit finite element model. Subsequently, the paravalvular regurgitant volume was estimated using a computationally efficient PVL model based on a thin-film approximation (Shvarts and Yastrebov 2018). This PVL model was successfully validated against both high-fidelity CFD simulations and experimental measurements. To enable comparison with their results, a deployment simulation was performed using the average male geometry from their study. In accordance with their simulation setup, a frame height of 45 mm, skirt height of 13 mm, and implantation depth of 9 mm were used. The remaining model parameters were varied within the ranges reported in Table 2. For the PVL calculation, the pressure waveforms shown in Figure 7 were scaled such that the mean transvalvular pressure difference during diastole was 100 mmHg and the diastolic duration was 0.56 s, consistent with the pressure conditions used in their simulations. Spanjaards et al. (2024) performed simulations with three stent sizes, referred to as small (26 mm), medium (29 mm), and large (31 mm). The annulus diameter of the average male geometry was 27 mm, which corresponds to the large stent (Piazza et al. 2012). Therefore, the paravalvular regurgitant volume predicted by our fast-to-evaluate model was compared with the result obtained with the large stent in their high-fidelity deployment simulation.

## Results

### In silico trial and validation

To assess the feasibility of conducting large-scale in silico TAVI trials, an example in silico trial was performed using the fast-to-evaluate model and the VCG. Deployment and leakage simulations were conducted for 500 virtual aortic stenosis geometries. For each geometry, between 45 and 63 simulations were completed, with one outlier having only 28 successful simulations. In total, 28,996 successful simulations were performed, which is 92% of all performed simulations. For each patient, the median RF across all simulations was calculated to obtain a single representative RF value. The resulting cohort mean was 19% ± 21%, compared with 16% ± 13% reported in a clinical study of TAVI patients (Crouch et al. (2015)). These values are of similar magnitude, indicating that the simulated RF values are consistent with the range observed clinically. In this section, we analyse how variations in model parameters and anatomical features influence the amount of PVL, quantified by RF.

**Fig. 8:**
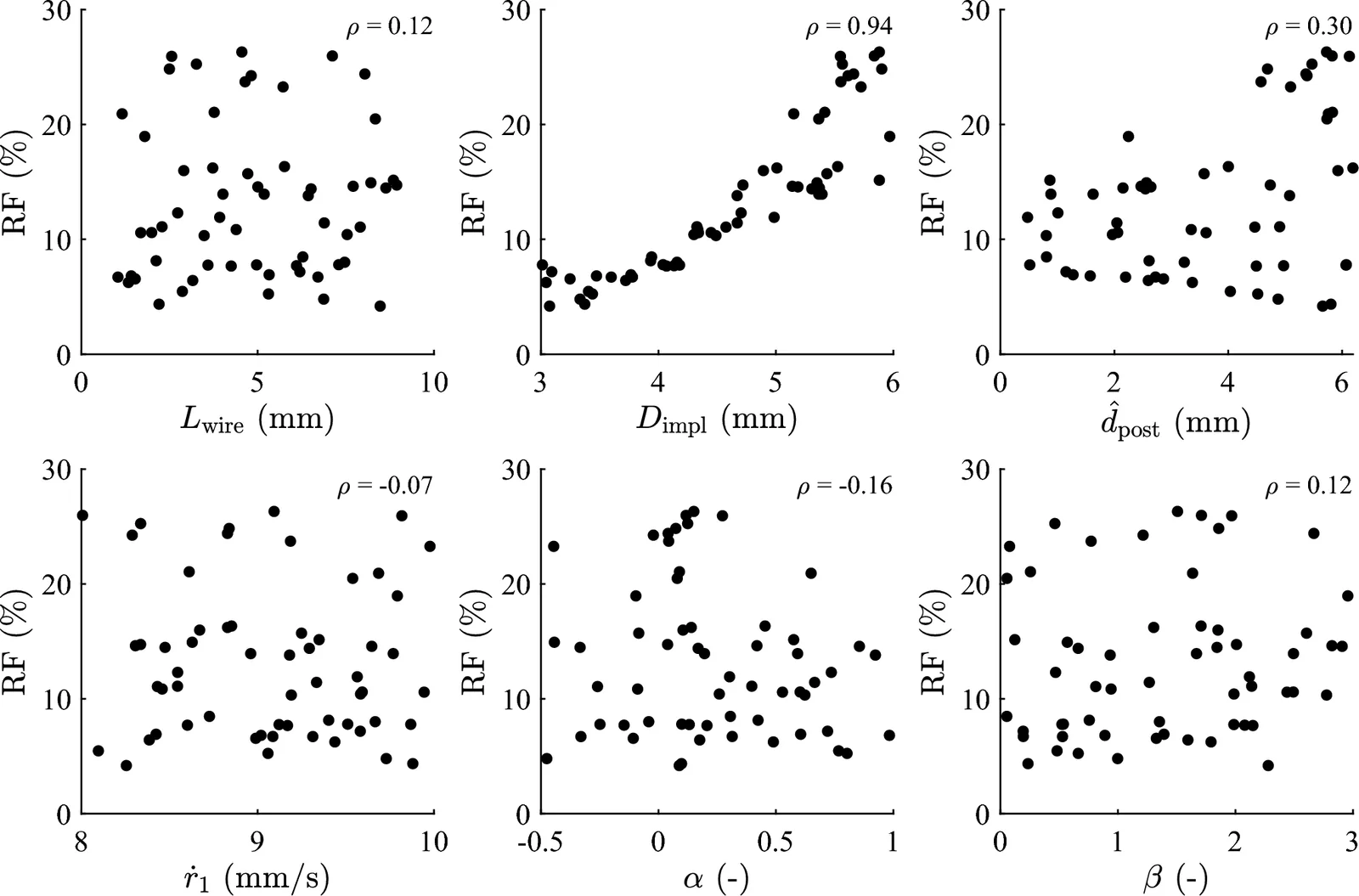
Regurgitant fraction (RF) as a function of the six varied model parameters, for the case whose median RF was closest to the overall median. Spearman correlation coefficients are depicted in the upper right corner

Figure 8 presents the results of an example virtual patient, whose median RF closely matched the population median. The figure illustrates RF as a function of the six varied model parameters. Among these, no clear correlations with RF were observed, except for the implantation depth ($D_{\text {impl}}$), which exhibited a strong positive correlation with RF (Spearman correlation coefficient: 0.94). This indicates that deeper implantation of the stent below the annulus plane was associated with increased paravalvular leakage.

**Fig. 9:**
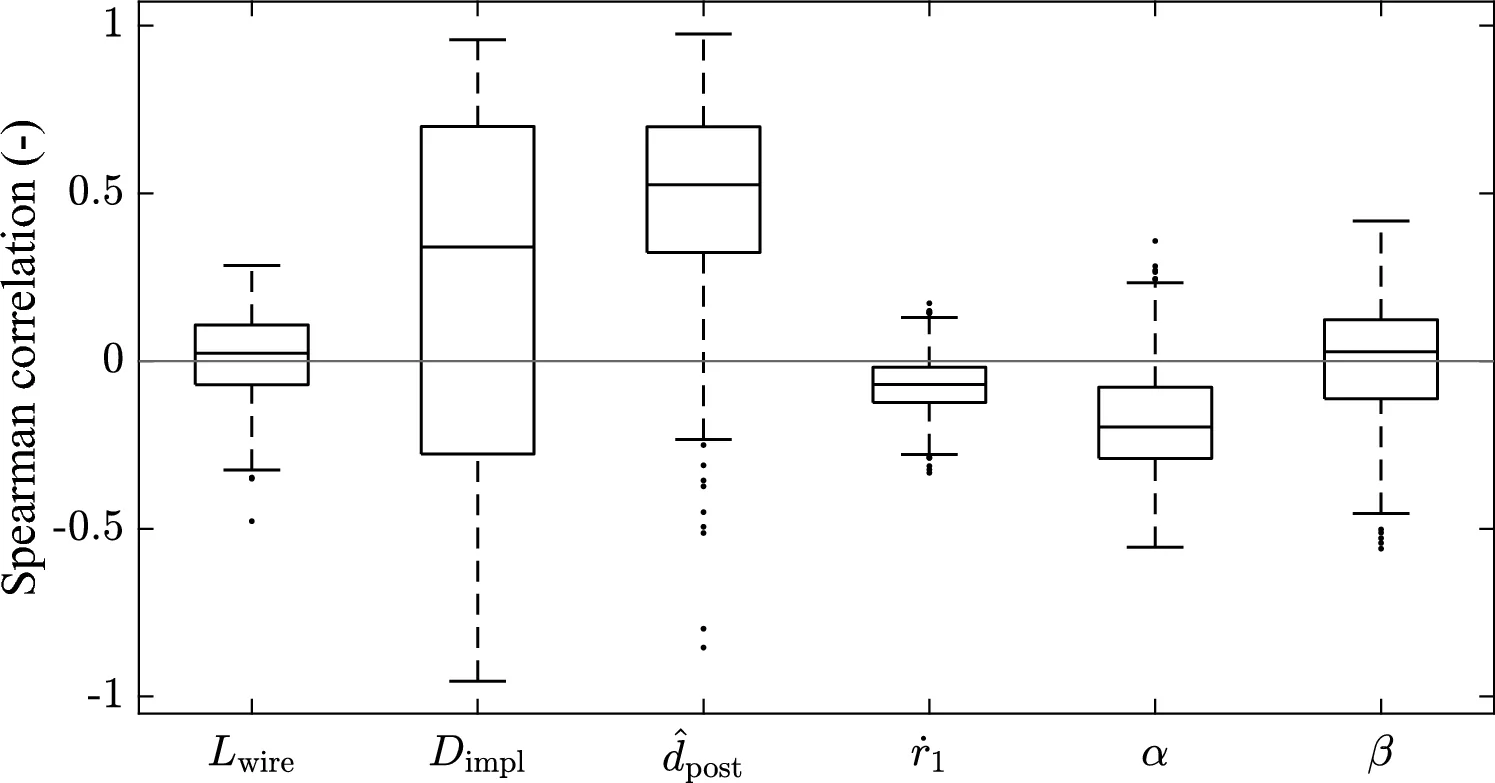
Boxplots of Spearman correlation coefficients between each of the six model parameters and regurgitant fraction

Figure 9 presents the distribution of Spearman correlation coefficients between RF and each of the six model parameters across all 500 virtual patients. A wide range of correlations was observed between implantation depth and RF, varying from strongly positive, as seen in Fig. 8, to strongly negative. This variability indicates that there was no consistent global relation, but relationship between implantation depth and RF appeared to be patient-specific. In contrast, a consistent positive correlation was observed between RF and the final distance between the native leaflets and the sinus wall ($\hat{d}_{\text {post}}$). This finding was expected, as a smaller post deployment distance allows for greater stent expansion, thereby reducing PVL. The remaining model parameters did not exhibit strong correlations with RF across the virtual cohort.

**Fig. 10:**
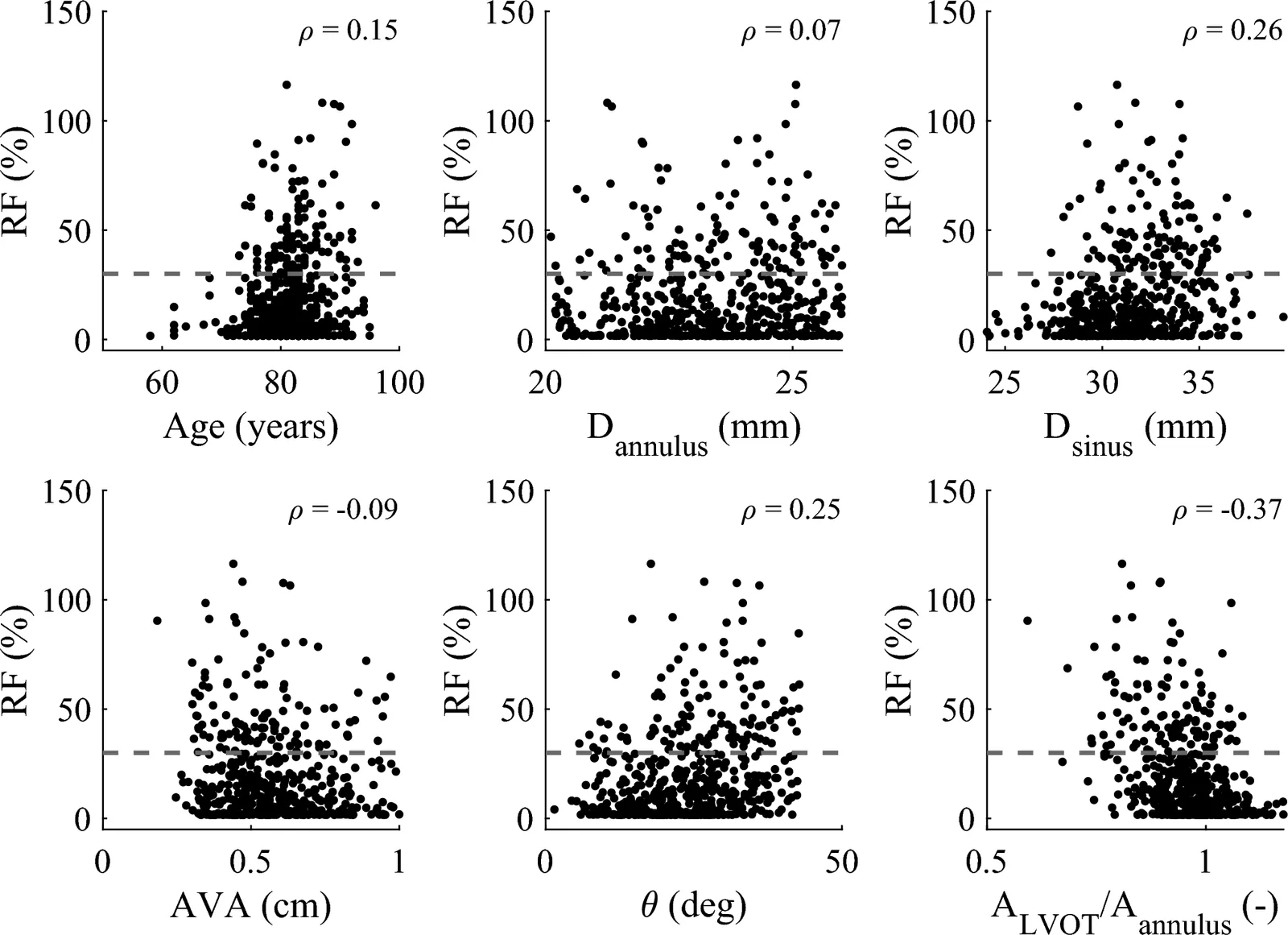
Median regurgitant fraction (RF) per patient as a function of age, and shape features. Spearman correlations coefficients are depicted in the upper right corner

Figure 10 illustrates the relationship between RF and patient age, and five different shape features. Contrary to expectations based on findings of Sherif et al. (2010), the correlation between RF and the angle between LVOT and ascending aorta (θ) was weak, with a Spearman correlation coefficient of only 0.25. A weak negative correlation (−0.37) was observed between RF and the ratio of LVOT to annulus area. No strong correlations were observed for the other shape parameters or age.

**Table 3:** Comparison of the non-significant (AR < 2) and significant (AR ≥ 2) aortic regurgitation between the clinical study by Sherif et al. (2010) and the in silico trial

	Clinical study (n=50)		In silico trial (n=500)	
	AR<2 (60%)	AR≥2 (40%)	AR<2 (77%)	AR≥2 (23%)
Age (years)	80.50 ± 7.99	80.55 ± 7.83	80.99 ± 5.52	82.87 ± 4.72
$D_{\text {annulus}}$ (mm)	23.50 ± 1.48	22.90 ± 1.25	23.31 ± 1.49	23.61 ± 1.47
$D_{\text {sinus}}$ (mm)	29.73 ± 3.40	29.75 ± 3.41	31.13 ± 2.53	32.54 ± 1.98
AVA (cm$^2$)	0.64 ± 0.18	0.64 ± 0.16	0.58 ± 0.16	0.54 ± 0.17
θ (deg)	15.70 ± 4.71	26.60 ± 8.19	23.40 ± 8.58	27.29 ± 9.08

Age and shape features are reported in mean ± standard deviation

**Fig. 11:**
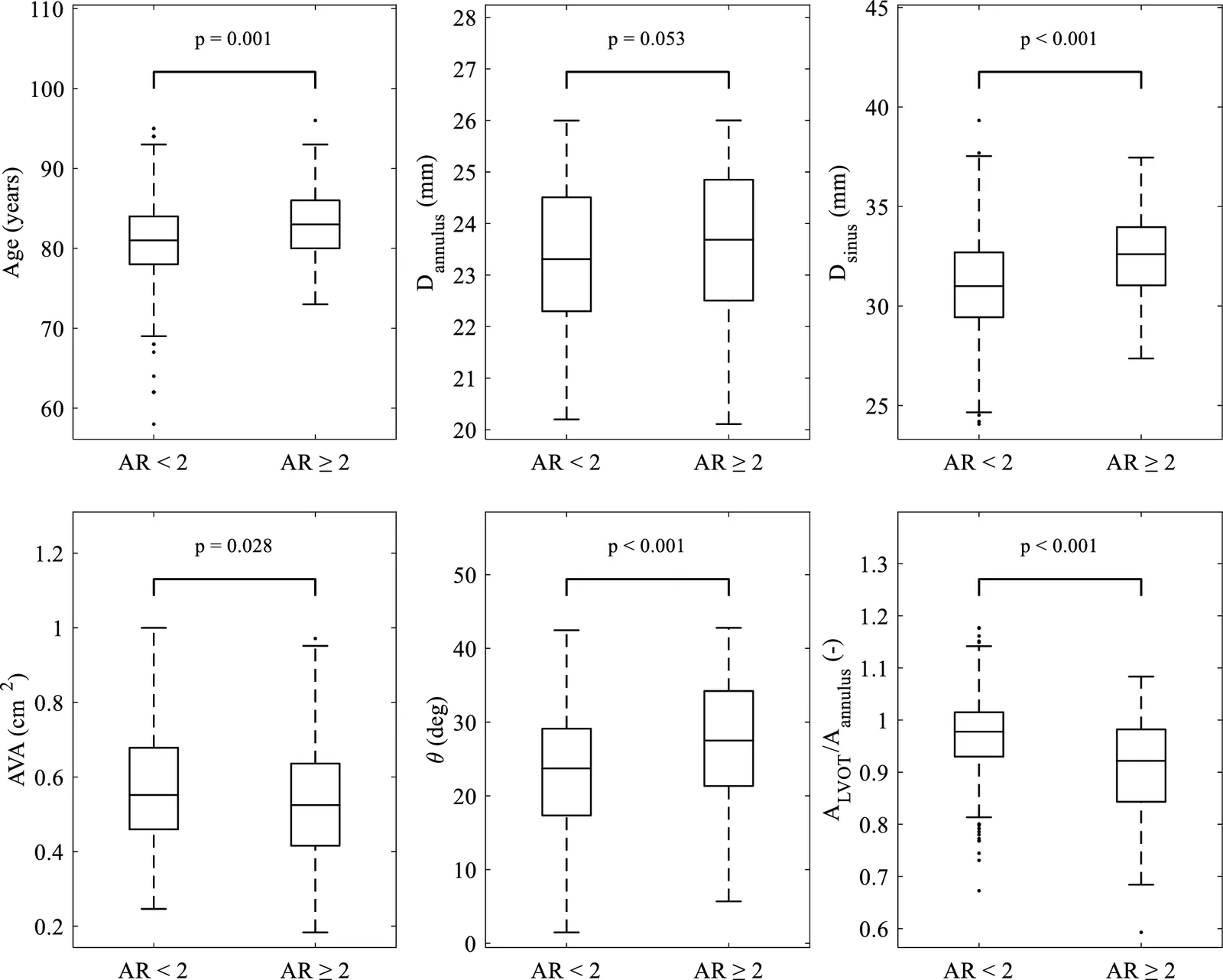
Boxplots of age and five shape features for non-significant (below grade II) and significant aortic regurgitation (grade II or higher) groups. P-values for the Wilcoxon rank sum test are indicated at the top

To enable direct comparison with the results found by Sherif et al. (2010), the virtual cohort was split into two groups based on the severity of aortic regurgitation (AR): significant AR (grade II or higher) and non-significant AR (below grade II). The median RF per patient was used for classification, where RF of 30% or higher corresponded to grade II or higher. Although Fig. 10 did not reveal strong correlations, some statistically significant differences emerged between the groups (Fig. 11). Wilcoxon rank-sum tests rejected the null hypothesis of equal medians for all characteristics except annulus diameter. The most prominent finding was that the angle θ was significantly larger in the significant AR group. Additionally, the significant AR group exhibited lower LVOT to annulus area ratios. A slightly larger sinus diameter was observed as well in this group.

The results of the in silico trial were compared with the findings from the clinical study of Sherif et al. (2010) in Table 3. In the virtual cohort 23% of the patients exhibited AR of grade II or higher, compared to 40% in the clinical study. While age, annulus diameter, sinus diameter, and AVA were nearly identical between the significant and non-significant AR groups in the clinical study, small differences were observed in the virtual cohort. Nevertheless, the values remained close to those reported clinically. On the contrary the difference in angle θ was more pronounced in the clinical study than in the in silico trial. However, both studies consistently showed that patients with significant AR had larger angles.

**Fig. 12:**
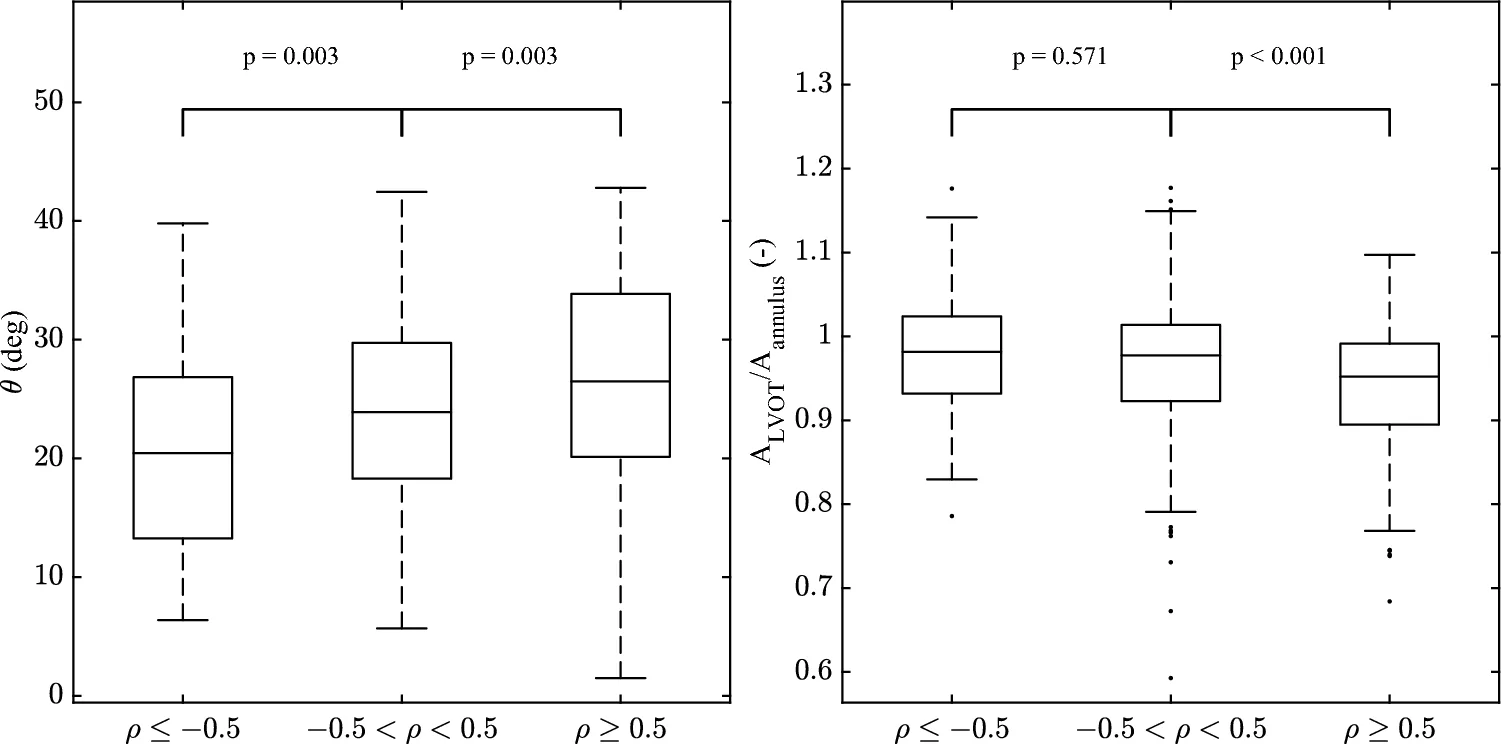
Boxplots of the LVOT-to-ascending aorta angle (left), and the ratio of LVOT to annulus area (right) for three different groups based on Spearman correlation (ρ) between implantation depth and RF

The wide variability in correlations between RF and implantation depth observed in Fig. 9 prompted further investigation into the underlying causes. As an additional analysis step, the virtual cohort was divided into three groups based on the Spearman correlation coefficient (ρ) between RF and implantation depth: strong negative correlation $\rho \le -0.5$, weak or no correlation -0.5<ρ<0.5, and strong positive correlation ($\rho \ge 0.5$). The goal was to identify whether specific anatomical features could explain the differing dependencies on implantation depth across patients. Two interesting differences were observed and shown in Fig. 12. First, the angle (θ) appeared to increase progressively across the three groups, from low angles in the strong negative correlation group to higher angles in the strong positive correlation group. This finding suggests that for patients with a larger angle, a shallower implantation depth may be more beneficial, whereas for those with a smaller angle, deeper implantation may reduce PVL. A Wilcoxon rank-sum test confirmed that the differences in angle between the groups were statistically significant. Second, the group with strong positive correlations also exhibited lower LVOT-to-annulus area ratios, indicating a more converging LVOT shape. This implies that patients with such geometries may benefit from shallower implantation depths. In contrast, no clear differences in this ratio were observed between the strong negative and weak correlation groups. These findings highlight that the optimal implantation depth is highly patient-specific and strongly influenced by individual aortic valve geometry.

### Comparison with high-fidelity model

A total of 42 successful simulations with different parameter combinations were performed with the fast-to-evaluate model using the average male geometry from Spanjaards et al. (2024). The fast-to-evaluate model predicted a paravalvular regurgitant volume of 11±1 mL, compared with 13 mL obtained for the large stent in the high-fidelity modelling framework of Spanjaards et al. (2024). Thus, the regurgitant volume predicted with the fast-to-evaluate model was comparable to that obtained with the high-fidelity model.

## Discussion

This study aimed to assess the feasibility of conducting large-scale in silico TAVI trials using the VCG developed by Verstraeten et al. (2024) in combination with a novel fast-to-evaluate model, for conducting in silico TAVI trials. More specifically, the objective was to investigate procedural and anatomical predictors of PVL after TAVI, as was done in an earlier clinical study by Sherif et al. (2010), and compare our in silico results to their clinical outcomes.

The VCG was extended with three new functionalities: gender-specific cohort generation was enabled, age was incorporated, and shape feature filtering options were extended. This extension enabled generating a virtual cohort that matched the clinical cohort in terms of male/female ratio and distributions of, annulus diameter, sinus diameter, AVA and the angle between LVOT and ascending aorta θ. Leveraging the scalability of the VCG, we created a cohort of 500 virtual patients, ten times larger than the original clinical cohort. Using this virtual cohort, TAVI deployment and PVL simulations were conducted with the novel fast-to-evaluate model, that is capable of evaluating 350 simulations per hour on a standard desktop computer using a single core. We successfully performed 45 to 63 simulations per patient, yielding nearly 29,000 simulations in total, a significant increase compared to previous studies on TAVI deployment simulations that typically conducted up to 60 simulations (Luraghi et al. 2019; De Jaegere et al. 2016; Bianchi et al. 2019; Mao et al. 2018; Spanjaards et al. 2024; Lavon et al. 2019).

For each virtual patient, i.e. aortic stenosis geometry, approximately 50 different combinations of model parameters were evaluated to assess their impact on PVL, quantified by the regurgitant fraction. The ability to perform multiple test deployments per patient, highlights another advantage of in silico trials compared to clinical studies. Among the model parameters tested, those without direct clinical meaning, i.e. $L_{\text {wire}}$, $\dot{r}_1$, α and β (Fig. 4, and Eq. 6) showed weak correlations with RF. Since these parameters are difficult to measure and control in clinical practice, this is a positive finding. In contrast, implantation depth exhibited a wide range of correlations, from strongly negative to strongly positive. Sherif et al. (2010), found a quadratic relationship between implantation depth and AR with a optimal depth of 9.5 mm. Since the LVOT lengths of the virtual aortic valve geometries were limited to 5 mm, we could not simulate this implantation depth and thus could not confirm the same optimum. Nevertheless, we did find that the relationship between RF and implantation depth is highly patient-specific, consistent with variability reported in other clinical studies (Hagar et al. 2020; Athappan et al. 2013; Takagi et al. 2011).

Further analysis revealed that higher angles θ were associated with stronger positive correlations between RF and implantation depth. This finding suggests that for patients with a large θ, shallower implantation may be preferable, whereas deeper implantation may benefit those with smaller angles. Additionally, positive correlations were more frequently observed in patients with converging LVOT shapes, implying that deep implantation may result in more AR in these aortic valve anatomies.

We also explored correlations between RF and various anatomical shape features, but found no strong correlations (all absolute correlation values < 0.5). However, when splitting the virtual cohort by AR severity (grade II or higher versus below grade II), differences emerged in age, sinus diameter, AVA and LVOT-to-annulus area ratio. These differences were not observed in the clinical study, potentially due to the limited sample size (50) from which the underlying correlations might be less evident. Moreover, the proportion of grade II or higher cases differed between the clinical (40%) and the virtual (23%) cohorts, suggesting lower PVL incidence in the latter. Despite the discrepancies, feature ranges in both groups in the in silico trial remained within the bounds of the corresponding clinical groups. Moreover, differences in θ between AR groups were consistent across both studies: 16$^\circ$ versus 27$^\circ$ in the clinical study and 23$^\circ$ versus 27$^\circ$ in the in silico trial, though the difference was smaller in the latter. As discussed by Verstraeten et al. (2024), this discrepancy may be attributed to differences in measurement techniques: Sherif et al. (2010) used 2D angiography, while our study employed 3D centerline-based angle calculations. Another notable observation was that the median LVOT-to-annulus area ratio in the AR < 2 group was close to 1, while it was lower in the significant AR group. This observation aligns with literature indicating that tubular LVOT shapes (i.e., straight tubes) are associated with reduced PVL compared to converging or diverging geometries (Condado et al. 2017).

As a first step towards model verification, the fast-to-evaluate model was compared with the high-fidelity frame work of Spanjaards et al. (2024). The resulting agreement in predicted paravalvular regurgitant volume (11 ± 1, versus 13 mL) provides initial support for the ability of the fast-to-evaluate model to reproduce PVL estimates of a model with higher fidelity. However, this comparison should not be considered as a complete verification of the model, as Spanjaards et al. (2024) explicitly included patient-specific calcifications, whereas calcifications were not represented in our model. Consequently, differences between the two models cannot solely be attributed to differences in model fidelity. Moreover, the comparison was limited to a single combination of geometry and pressure boundary conditions. Further verification across a broader range of anatomies and hemodynamic conditions is therefore required to completely verify the model.

### Limitations and future work

Although the findings regarding PVL in the in silico trial aligned well with those from the clinical study by Sherif et al. (2010) and other clinical studies, supporting the feasibility of using virtual cohorts in in silico TAVI trials, several limitations should be acknowledged.

The first limitation concerns the trade-off between computational efficiency and model accuracy. By employing a fast-to-evaluate model, we were able to simulate large-scale in silico trials, but this came at the cost of reduced precision. High-fidelity models such as CFD or FSI models, as used by De Jaegere et al. (2016) and Luraghi et al. (2019) provide more accurate PVL estimations. In our study, the reduced accuracy may have contributed to the median regurgitant fraction exceeding 100% in four virtual patients (0.8% of the cohort), which is physiologically implausible. Nevertheless, the computational efficiency of the fast-to-evaluate model, outweighs this limitation when the goal is to explore general trends and distributions across the virtual cohort. Moreover, the fast-to-evaluate model could be used to do a fast, large-scale screening for potential, relevant anatomical and procedural parameters. High-fidelity models could subsequently be used to confirm these findings. For patient-specific treatment planning or outcome prediction, however, higher model fidelity would be essential.

The second limitation concerns model simplifications that limit the applicability of the fast-to-evaluate model. The most important simplification in the context of PVL prediction is that calcifications are not explicitly modelled. The presence of calcification deposits can strongly affect device deployment and sealing and may therefore strongly influence PVL (Spanjaards et al. 2024). However, the VCG and the fast-to-evaluate model both currently do not support local calcification deposits. Therefore, the this study focuses on morphological and procedural factors affecting PVL. Nevertheless, the mechanical effects of calcification could be modelled to some extent by increasing leaflet stiffness of one or more leaflets. However, translating the amount, morphology, and spatial distribution of calcification into an effective leaflet stiffness would require a dedicated calibration study, which is beyond the scope of the present work. Extending both the VCG and the fast-to-evaluate model to include spatially varying calcification patterns, as was done in the studies of Oldenburg et al. (2024) and Spanjaards et al. (2024) would therefore be an important direction for future work.

In addition to the absence of calcifications, the current NURBS template consists of three leaflets and therefore cannot represent bicuspid aortic valves. Moreover, the leaflets in the NURBS template are symmetric, and this symmetry is retained after fitting the template to the patient-specific geometries. Consequently, highly asymmetric or irregular leaflet anatomies cannot be accurately represented. Additionally, the current deployment model was developed for self-expanding TAVI devices and cannot be directly applied to balloon-expandable devices. These simplifications restrict the current applicability and accuracy of the model to tricuspid aortic valves with relatively regular and homogenous leaflets treated with self-expanding devices.

Another limitation relates to the translation of the clinical oversizing into the deployment model. In vivo, the aortic root wall deforms under radial forces from the stent, but the fast-to-evaluate model did not account for the material properties of either the stent or the aortic wall. To avoid overestimating PVL, we allowed the expanding cylinder to exceed the aortic diameter. Sensitivity analysis revealed that the model was highly sensitive to this “oversizing” parameter. To be able to investigate the effect of the other model parameters, we therefore opted to fix it at 3 mm, corresponding to the minimum oversizing used clinically with the Medtronic CoreValve. This choice may have contributed to the lower incidence of significant AR observed in the in silico trial. Future research should focus on further testing, optimisation and calibration of the fast-to-evaluate model to determine an appropriate value for the oversizing parameter.

Beyond these limitations in model fidelity, the scope of the presented in silico trial was restricted to PVL as the only post-procedural complication. In clinical practice, procedural parameters that reduce PVL may simultaneously increase the risk of other complications. For instance, valve oversizing (i.e. the prosthetic valve diameter being too large compared to the patient’s annulus diameter), can lead to excessive radial expansion forces on the aortic wall. These forces can cause conduction tissue damage (Hopf et al. 2017; Finotello et al. 2017), and increases the risk of the need for permanent pacemaker implantation (Rudolph et al. 2023). Similarly, several studies found that implantation depth influences the risk of conduction disturbances, and the need for pacemaker implantation (Piazza et al. 2016; Breitbart et al. 2021). If the goal of an in silico trial is to minimise PVL alone, focusing exclusively on PVL-related metrics may be sufficient. However, if the aim is to optimise the device for overall patient benefit and to minimise the risk of all complications, additional outcome metrics, such as conduction disturbances, should be incorporated into the model.

Long-term durability is another important aspect that cannot be directly assessed with the fast-to-evaluate model. Structural valve degeneration, for example, is a complex process that occurs over much longer timescales than those considered in the model. However, the model can identify deployment-related issues that may contribute to or accelerate structural valve degeneration, such as PVL and prosthesis malpositioning (Rauseo et al. 2026). These issues could adversely affect long-term valve durability.

Addressing these limitations will require further development of both the VCG and the fast-to-evaluate model. To improve the applicability of the virtual cohort generator in in silico trials, the VCG should be extended to include post-TAVI boundary conditions, enabling the use of patient-specific boundary conditions. Moreover, the fast-to-evaluate model could be made more patient-specific by incorporating variable leaflet stiffness based on the amount of calcification per leaflet.

Finally, further calibration and verification of the fast-to-evaluate model are required before it can be used for device design optimization. In particular, important model parameters such as leaflet stiffness to account for calcifications and oversizing parameter, should first be calibrated against high-fidelity deployment simulations. the resulting calibrated model should subsequently be verified further against independent high-fidelity simulations across a range of anatomies, and procedural settings. Once its strengths and limitations are well characterised, the model could even support patient-specific treatment planning and outcome prediction. For such applications, its computational efficiency offers a great advantage over computationally expensive high-fidelity models, especially when multiple procedural scenarios must be evaluated to optimise the treatment for an individual patient.

## Conclusion

This study assessed the feasibility of conducting large-scale in silico trials using virtual cohorts generated by the VCG in combination with a novel fast-to-evaluate model to conduct large-scale in silico TAVI trials. The developed fast-to-evaluate model enabled investigation of nearly 29,000 TAVI deployment scenarios across 500 virtual patients. Anatomical and procedural predictors for PVL were investigated, and similar findings were found compared to the clinical study of Sherif et al. (2010). Importantly, the relationship between the LVOT-to-ascending aorta angle and regurgitant fraction observed in the in silico trial was consistent with clinical findings, supporting the validity of the in silico trials. Additional correlations between paravalvular leakage and anatomical or procedural features also aligned with trends reported in other studies. The ability to systematically test multiple TAVI configurations per patient, something not feasible in clinical practice, offers valuable insights for device design and optimisation. Overall, the results demonstrate the feasibility of large-scale in silico TAVI trials and highlight the potential of combining the VCG with the novel fast-to-evaluate model as a tool for TAVI manufacturers to support device development and improvement.

## Data Availability

The data that support the findings of this study are openly available in 4TU.Research Data at https://doi.org/10.4121/4a1cce8a-0dfe-455b-b00d-3f55925572c2.

## Abbreviations

AR: Aortic regurgitation
AVA: Aortic valve area
CFD: Computational fluid dynamics
FEA: Finite element analysis
FSI: Fluid–structure interaction
LV: Left ventricle
LVOT: Left ventricular outflow tract
NURBS: Non-uniform rational B-splines
PVL: Paravalvular leakage
RF: Regurgitant fraction
RV: Regurgitant volume
SEV: Self-expanding valve
STL: Stereolithography
SV: Stroke volume
TAVI: Transcatheter aortic valve implantation
VCG: Virtual cohort generator
